# Hierarchical Hybrid Coatings with Drug-Eluting Capacity for Mg Alloy Biomaterials

**DOI:** 10.3390/ma16247688

**Published:** 2023-12-18

**Authors:** Ana Nicolao-Gómez, Enrique Martínez-Campos, Lara Moreno, Juan Rodríguez-Hernández, Endzhe Matykina

**Affiliations:** 1 Departamento de Ingeniería Química y de Materiales, Facultad de Ciencias Químicas, Universidad Complutense, 28040 Madrid, Spain; anicolao@ucm.es (A.N.-G.); laramo01@ucm.es (L.M.); 2 Funcionalización de Polímeros (FUPOL), Instituto de Ciencia y Tecnología de Polímeros (ICTP-CSIC), 28006 Madrid, Spain; e.martinez.campos@csic.es (E.M.-C.); jrodriguez@ictp.csic.es (J.R.-H.); 3 Unidad Asociada al ICTP-CSIC, Instituto de Química Médica (IQM-CSIC), Grupo de Síntesis Orgánica y Bioevaluación, Instituto Pluridisciplinar (UCM), Paseo de Juan XXIII 1, 28040 Madrid, Spain

**Keywords:** magnesium, implant, plasma electrolytic oxidation, polycaprolactone, corrosion, drug delivery

## Abstract

A hierarchical hybrid coating (HHC) comprising a ceramic oxide layer and two biodegradable polymeric (polycaprolactone, PCL) layers has been developed on Mg3Zn0.4Ca cast alloy in order to provide a controlled degradation rate and functionality by creating a favorable porous surface topography for cell adhesion. The inner, ceramic layer formed by plasma electrolytic oxidation (PEO) has been enriched in bioactive elements (Ca, P, Si). The intermediate PCL layer sealed the defect in the PEO layer and the outer microporous PCL layer loaded with the appropriate active molecule, thus providing drug-eluting capacity. Morphological, chemical, and biological characterizations of the manufactured coatings loaded with ciprofloxacin (CIP) and paracetamol (PAR) have been carried out. In vitro assays with cell lines relevant for cardiovascular implants and bone prosthesis (endothelial cells and premyoblasts) showed that the drug-loaded coating allows for cell proliferation and viability. The study of CIP and PAR cytotoxicity and release rate indicated that the porous PCL layer does not release concentrations detrimental to the cells. However, complete system assays revealed that corrosion behavior and increase of the pH negatively affects cell viability. H_2_ evolution during corrosion of Mg alloy substrate generates blisters in PCL layer that accelerate the corrosion locally in crevice microenvironment. A detailed mechanism of the system degradation is disclosed. The accelerated degradation of the developed system may present interest for its further adaptation to new cancer therapy strategies.

## 1. Introduction

In order to fulfill current biomedical challenges, a large body of research has been focused in the last decade on the design of hybrid materials that consist of a combination of a metallic core with ceramic and polymer coatings [1]. Mg alloy based biodegradable biomaterials are being developed for temporary implant applications in orthopedics and angioplasty. However, Mg alloys biodegradability dynamics are affected by corrosion processes, which may lead to a premature loss of implant integrity [2]. On the other hand, it has recently been demonstrated that corrosion process of Mg and the pH increase of the surrounding environment as a result of the H_2_ gas generation (Equation (1)) inhibits proliferation of cancer cells, e.g., osteosarcoma, among others [3].

Mg + 2H_2_O → Mg^2+^ + H_2g_ + 2OH^−^ (1)

Synchronization of implant degradation with tissue regeneration is, therefore, a key requirement in implantation and therapy procedures.

Plasma electrolytic oxidation (PEO), also known as micro-arc oxidation (MAO), is a surface treatment applicable to conventional and additively manufactured light alloys [4]. Mg alloys produce ceramic MgO-based coatings that act as a barrier, delaying the onset of corrosion process in the Mg substrate [5]. These coatings are intrinsically porous, which is beneficial for cell-implant interaction but impairs their protective capacity. As a result, hybrid coating systems have emerged that focused on formation of dense polymer pore-sealing layers in order to improve the corrosion resistance of ceramic coated Mg alloys [6,7,8]. The studies described above show little concern for the surface topography of the polymer layer [9,10]. However, a proper control of the surface structure on a micrometric scale, together with the chemical aspects, can significantly affect the bioactivity of the material.

Cell-biomaterial interaction is determined mainly by relevant surface properties such as stiffness, ionic charge, hydrophilicity, or surface topography (including the formation of surface microstructures or porous interfaces). Controlling the topography of a polymeric top-coat of a hybrid hierarchical material is a key feature for enhancing cell processes, such as adhesion, proliferation, or differentiation [11]. One of the most extensively used procedures to form an ordered porous surface is the Breath Figures (BF) method. This method is based on the evaporation of a volatile solvent from a polymer solution at high relative humidity (typically RH > 70%). The moisture condenses on the cold surface of the material, forming water droplets, which grow during the evaporation of the solvent, giving rise to pores [12]. The porous morphology of polymer layer provides a topography that favors cell adhesion, proliferation, and differentiation, as it mimics important microtopographical features of the native extracellular matrix. As seen in previous studies, the BF technique can form a fully permeable hierarchical scaffold that can improve cell migration and colonization [13]. Proper molecular signaling can control cell proliferation and migration, modulate immune system response, or reduce bacterial infections, facilitating implantation success.

Another interesting feature of these systems is the possibility of loading drugs, molecules, or growth factors into the polymer layer that could, in turn, be released in a controlled manner [14,15]. The pharmaceuticals that have been studied in relation to drug-eluting Mg implants include anti-inflammatory agents (ibuprofen) [16,17], antibiotics (doxycycline, amoxicillin, vancomycin, gentamicin) [18,19], and cell proliferation inhibitors (sirolimus or paclitaxel) [20,21,22], the latter being relevant to attenuation of restenosis in cardiovascular stent applications. The microporous polymer layer, obtained by the BF approach enabled drug-eluting feature, also renders Mg alloys an interesting alternative for orthopedic implants in osteosarcoma patients requiring tumorigenic tissue removal [11]. The implant can be loaded with specific chemotherapeutic agents such as methotrexate, doxorubicin, and cisplatin [23]. Localized drug release minimizes adverse effects on healthy tissues and optimizes drug activity [24]. Additionally, combining anti-cancer drugs with the hydrogen (H_2_) generation from the magnesium corrosion process may enhance the eradication of residual osteosarcoma cells [25]. This approach also may prove beneficial in addressing cancer bone metastatic osteolysis, wherein anti-osteoclast drugs like bisphosphonates or denosumab can be adjunctively utilized to mitigate osteolysis [26].

In a hierarchical material, drugs can be loaded in each layer depending on the final desired effect. For example, the kinetic of the drug release can be controlled by the size and morphology of the pores of the ceramic layer [27]. On the other hand, the thickness, the permeability, and the degradation rate of the polymeric layer can affect drug release behavior [28]. In addition, drugs with low solubility can be transported in hydrophobic polymeric matrices or encapsulated in porous layers, thereby reducing the burst release effect occurring at short times and thus reducing their toxicity [29]. Investigating the effect of the BF morphologies of the polymer layer, Ponnusamy et al. have shown that faster release rates of salicylic acid and ibuprofen are achieved from porous PLGA and (PEG)/PLGA BF films compared to monolithic polymer films [30]. This suggests that use of BF microporous polymeric top layers as part of a hybrid system is worth exploring as it may offer an opportunity for additional tuning of the drug release rate.

The present hybrid hierarchical coating (HHC) system, comprising a ceramic PEO layer and a BF polycaprolactone (PCL) layer, has been developed as an example of such tailored design. In a previous work, a hierarchical biodegradable polymer layer was shown to improve the corrosion performance of PEO coatings on Mg alloys by infiltrating and sealing their intrinsic pores and cracks [31]. It has further been shown that ciprofloxacin loaded into the BF PCL top-coat of this HHC system acts as an active corrosion inhibitor [32].

In this work, the characterization and evaluation of this system functionalized with model drugs (N-acetyl-para-aminophenol, better known as acetaminophen or paracetamol, and ciprofloxacin) is carried out in terms of biological responses to drug release kinetics. The main goal is to determine the degree of the cytotoxicity that a model Mg3Zn0.4Ca alloy implant with loaded HHC system may cause as a result of a drug release and the corrosion of magnesium substrate on mouse premyoblast and endothelial cells. The latter are examples of healthy tissues relevant to Mg implant applications in treatment of cardiovascular system diseases, cancer therapies, or as prostheses for bone fracture treatments.

## 2. Materials and Methods

### 2.1. Materials

Mg3Zn0.4Ca cast alloy (mass fraction: 0.4% Ca; 0.012% Fe; 0.0015% Cu; 3.14% Zn and Mg balance) was selected as substrate. Cast ingots were supplied by Helmholtz-Zentrum Hereon, Institute of Surface Science (Geesthacht, Germany). The ingots were cut into 10 × 10 × 4 mm specimens, which were successively ground on all sides with SiC abrasive papers to P1200 grit size, cleaned with deionized water and isopropyl alcohol, and dried in warm air prior to PEO treatment. 

PCL (PBI 010) was purchased from Natureplast (Mondeville, France). Paracetamol (further, PAR) and ciprofloxacin (further, CIP) were obtained from Thermo Scientific (Madrid, Spain).

### 2.2. PEO Treatment

The electrolyte for the PEO treatment was based on Ca, P, and Si species (9 g/L Na_2_SiO_3_·5H_2_O, 10 g/L Na_3_PO_4_·12H_2_O, 2.9 g/L CaO and 8 g/L KOH). The treatment was carried out for 300 s, using a 2 kW regulated AC power supply (EAC-S2000, ET Systems electronic, Altlußheim, Germany), with a square waveform peak to peak voltage of 400 V, a frequency of 50 Hz, and a current density limit of 100 mA·cm^−2^. After the PEO process, the specimens were rinsed in isopropanol and dried in warm air.

### 2.3. BF PCL Coating

**Figure 1:**
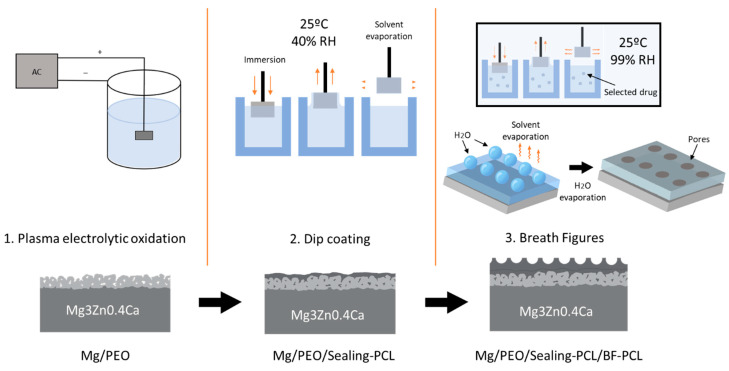
Schematic representation of the Mg-HHC system fabrication stages.

The deposition of the BF polymer layer was carried out using a dip coating technique (model VT-04 control unit) using a 75 mg/mL solution of PCL in chloroform under room temperature in a hermetic closed chamber. Figure 1 shows the general scheme followed in order to produce a complete PAR- or CIP-functionalized HHC system on a Mg3Zn0.4Ca alloy (further Mg-HCC). First, PEO is carried out in order to obtain a ceramic porous surface with controlled thickness. Second, the PEO coating layer is sealed by a thin planar PCL layer prepared by dip coating at low relative humidity (<40% RH). This intermediate layer ensures a good adhesion of the top-coat. During the last step, a thick, porous BF-PCL top-coat with a desired porous topography is formed at high relative humidity (98–99% RH) inside of a hermetic closed chamber by using a dip-coater.

Table 1 specifies the dip-coating process parameters for the sealing and top-coat PCL layers. Pharmaceutical agents were incorporated into the porous PCL top-coat layer by dissolving either PAR or CIP at 5 wt.% in the PCL-chloroform solution.

In order to reproduce only the top polymeric part of the HHC system, denominated as BF-PCL film, PCL films were developed on glass disks (Ø 12 mm) using a 30 mg/mL of PCL concentration. For this purpose, a 90 μL droplet of solution was deposited onto each disk and allowed to dry in a hermetic closed chamber with a moist atmosphere (98–99% RH). In the case of drug-loaded films, the same procedure was followed, introducing 5 wt.% of either PAR or CIP in a 30 mg/mL PCL/chloroform solution. The drug-loaded and blank BF-PCL films were used to discriminate the cytotoxicity of a drug from that of a combined effect of drug and Mg degradation.

### 2.4. Cell Culture

C2C12-GFP (ATCC CRL-1772, Manassas, VI, USA) mouse premyoblast cell line and C166-GFP (ATCC CRL-2583, Manassas, VI, USA) mouse endothelial cell line were incubated at 37 °C with 5% of CO_2_ in Dulbecco’s MEM complete medium (DMEM, D6429, Merck, Darmstadt, Germany), supplemented with 10% fetal bovine serum (FBS, Hyclone, Fisher Scientific, Madrid, Spain) and antibiotics (100 U/mL penicillin and 100 μg/mL streptomycin sulfate, Merck).

Complete biomaterial system specimens (Mg-HHC) were sterilized by 12 min of ultraviolet (UV) irradiation per each side of the specimen (72 min total) before cell assays. BF-PCL films received 45 min of UV irradiation per side. Each cell line was seeded over Mg-HCC at a density of 4 × 10^4^ cells per specimen, and over BF-PCL films at 2 × 10^4^ cells per film. 

Cell cultures were evaluated daily by fluorescence microscopy (FITC filter λex/λem = 490/525 nm) with an inverted fluorescence microscope (Olympus IX51), due to cells’ self-fluorescence. After 96 h of growth, cells were fixed with formalin and dehydrated by ethanol gradients of increasing concentrations for preservation and subsequent SEM study.

### 2.5. Drug Cytotoxicity

The cytotoxic effect of selected drugs in described cell models proliferating over TCP (tissue culture plastic) was analyzed to discern whether cellular response is caused by the drugs or by other elements of HHC system. Both cell lines were seeded at a density of 2 × 10^4^ cells per well, in a 12-well plate (Art. No. 150628, Thermo Scientific). C2C12-GFP and C166-GFP cells were left to grow for 48 h to reach a higher confluence degree. Subsequently, increasing concentrations of PAR or CIP (0 μg/mL, 50 μg/mL, 120 μg/mL and 200 μg/mL) were added to culture media and left for an additional 24 h. Micrographs were obtained of each condition; then, DNA quantification to determine cell death, and therefore cytotoxicity, was measured using a fluorescent reagent (FluoReporter^®^ Blue Fluorometric dsDNA Quantitation Kit, Art. No. F-2962, Fisher Scientific, Madrid, Spain). Each condition was measured in triplicate. The fluorescence of each well was measured with a plate reader (Synergy HT, BioTek, Winooski, VT, USA).

### 2.6. Cytocompatibility

To determine cell response to corrosion process of the Mg-HHC system, cell viability and proliferation were previously tested on functionalized BF-PCL films before analyzing cytocompatibility of the Mg-HHC specimens. Cell cultures were established as described in Section 2.4. After 96 h of growth, metabolic activity of premyoblastic and endothelial cells was quantified by Alamar Blue assay (alamarBlue^®^ Cell Viability Reagent, Art. No. DAL1025 and DAL1100), performed as described in previous studies [33] by adding 10% of the well volume in dark conditions and incubating for 1h 30 min at 37 °C. Fluorescence measurements (λex/λem 535/590 nm) were obtained with a plate reader (Synergy HT, BioTek). Total dsDNA was quantified using a fluorescent reagent (FluoReporter^®^ Blue Fluorometric dsDNA Quantitation Kit, Art. No. F-2962). Each condition was tested in triplicate.

A GLP22 pH meter (Crison) and a pH 52 30 electrode (Crison Barcelona, Alella, Spain) were used to perform the pH measurements of the culture medium. The pH changes in Mg samples experiments were analyzed removing 2 mL of medium from each well daily, which were then replaced with fresh complete DMEM.

### 2.7. Coating Characterization

Cross-sections and plan views of non-loaded and loaded Mg-HHC were examined using a JEOL JSM-820 (Tokyo, Japan) scanning electron microscope (SEM) equipped with an Oxford Link energy dispersive X-ray (EDS) microanalysis spectrometer. In addition, cultured Mg-HHC and Mg-HHC + CIP systems were characterized after four days of immersion in the DMEM solution using the JEOL JSM6400 instrument.

Metallographic preparation of the Mg-HHC cross-sections was carried out by grinding through SiC to P1200, followed by polishing with 3 μm and 1 μm diamond paste. 2D and 3D images of cultured Mg-HHC and Mg-HHC + CIP specimens were analyzed using a focus-variation 3D optical profilometer (InfiniteFocusSL, ALICONA, Graz, Austria), with ×50 lens. The same instrument was used to evaluate the surface roughness parameter S_a_ (arithmetical mean height of the area). The BF-PCL layer porosity was evaluated using a public domain image analysis software ImageJ 1.54 g (NIH, LOCI, University of Wisconsin, Madison, WI, USA).

### 2.8. Drug Release

PAR and CIP release from BF-PCL films and the Mg-HHC system was measured in modified α-MEM solution (prepared in the laboratory, free of all organic additives), following the immersion of the specimens in 4 mL of solution at 37 °C. At regular time intervals, a 2 mL aliquot of the test solution was withdrawn and, after measurement, returned to the system so as not to change the concentration of the medium. The drug concentration of the solution was measured using a UV-Vis spectrometer (PerkinElmer instrument, Lambda35, Waltham, MA, USA) in the range 200–400 nm in triplicate. PAR release was quantified at 245 nm and CIP at 320 nm.

PAR and CIP release was also measured from Mg-HHC specimens after 96 h of cell culture in DMEM solution at 37 °C. Each day, 2 mL of medium was collected from each well (containing 6 mL) and replenished with fresh medium. The concentration of PAR and CIP were measured with a plate reader (Synergy HT, BioTek) at 260 nm.

### 2.9. Statistical Analysis

Statistical analysis (mean ± standard deviation) of the triplicates’ resulting data was performed by means of an unpaired t-test, with a confidence interval of 95% (*p* < 0.05), with GraphPad *t*-test calculator (Dotmatics, Boston, MA, USA, https://www.graphpad.com/quickcalcs/ttest1/). Significant differences stand for * (*p* ≤ 0.05), ** (*p* ≤ 0.01), *** (*p* ≤ 0.001).

## 3. Results and Discussion

### 3.1. Coating Characterization

**Figure 2:**
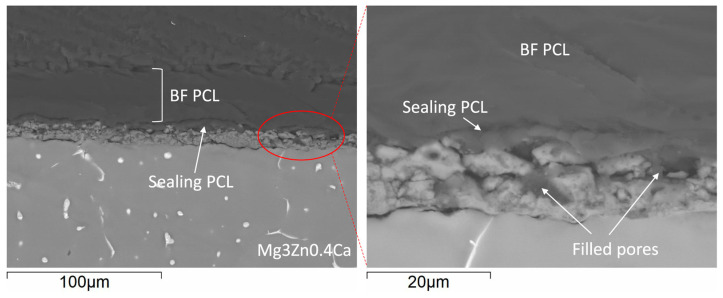
Cross-sectional backscattered electron micrographs of HHC system on Mg3Zn0.4Ca alloy.

The cross-section images of Mg-HHC (Figure 2) (i.e., samples treated following the procedure depicted in Figure 1) indicate the formation of a homogeneous coating with ~50 μm thickness, where both ceramic and polymer layers are clearly distinguished. The PCL sealing layer (3–4 μm thickness) penetrates the pores of the PEO coating and fills them, thus preventing the access of corrosive species from the medium. The outer layer is thick and contains surface pores.

**Figure 3:**
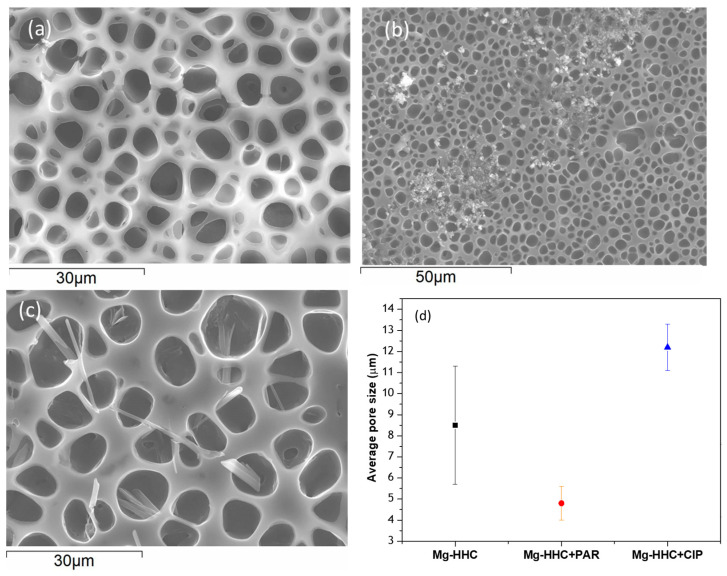
Secondary electron plan view micrographs of different Mg/PEO/Sealing-PCL/BF-PCL coating drug conditions: (**a**) BF-PCL without drug, (**b**) BF-PCL + paracetamol and (**c**) BF-PCL + ciprofloxacin; (**d**) average pore size variation.

According to the images shown in Figure 3, homogeneous pore distribution can be observed in as received Mg-HHC (Figure 3a) and drug loaded Mg-HHC (Figure 3b,c) systems. The incorporation of PAR and CIP influence the pore size (Figure 3d). The pores are smaller (6 μm) and larger (12 μm) in PAR- and CIP-loaded systems, respectively, in comparison with as-received one. PAR is incorporated as polygonal particles with different sizes either embedded or deposited on the surface of PCL (Figure 3b). The incorporation of CIP occurs in the form of needles penetrating the PCL layer (Figure 3c) where some of them protrude through the pores of the coating. These morphological differences may be attributed to the solubility of the drug in the PCL/chloroform solution, where CIP presents lower solubility compared to PAR.

### 3.2. Paracetamol and Ciprofloxacin Release from BF PCL Layer

**Figure 4:**
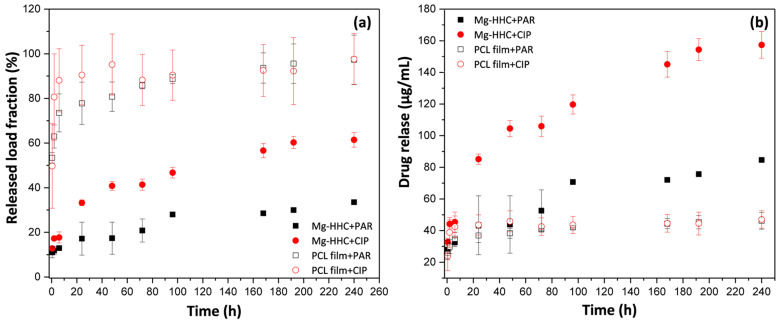
Released drug load fraction from BF PCL films and Mg-HHC system (**a**) and cumulative release from Mg-HHC system (**b**) over 10 days of immersion in inorganic alpha-MEM incubated at 37 °C.

The release profiles of CIP and PAR from BF-PCL films and loaded Mg-HHC system analyzed during 10 days of immersion in inorganic α-MEM solution are illustrated in Figure 4a. Important differences between the films and the complete HHC system were found. Specifically, higher PAR and CIP release from BF-PCL films were observed. BF-PCL films revealed a burst release during the first hour of immersion followed by a further release of the remaining load up to ~90% in 96 h. However, for the loaded Mg-HHC system, a rather gradual elution was observed over the time, with only 20 and 40% of PAR and CIP loads released after 10 days, respectively. This may be associated with the formation of insoluble chelates between Mg^2+^ and Ca^2+^ and CIP^0^ or PAR^0^ (Zwitterionic) in the medium [34,35,36,37], reducing the PAR and CIP release. This is in agreement with the observation of other previous reports where the complexation of different cations with CIP and PAR structures has been shown to reduce bioavailability [38,39]. According to the cumulative release concentration (Figure 4b), loaded Mg-HHC systems were reaching 70 and 120 μg/mL for PAR and CIP, respectively, after 96 h, which usually corresponds to the maximum period of cell culture experiments. Note that the cumulative drug release concentrations were lower for PCL-coated glass due to the fact that only one side of the glass disc (0.785 cm^2^) contained a drug; in case of a complete Mg-HHC system, the entire surface area (3.6 cm^2^) was loaded.

### 3.3. In Vitro Cytotoxicity Evaluation of Paracetamol and Ciprofloxacin

For bio-validation purposes, an analysis of potential cytotoxicity of drug-loaded implants, with respect to the surrounding tissues, was carried out. Therefore, in vitro assays were performed using cell lines representative of the tissues that could be in vivo affected by an eventual drug-release (muscle and endothelium). The effect that the addition of drugs may have on cellular response was first analyzed in TCP substrates in order to distinguish it. Then, in subsequent experiments the experiments were carried out with complete system (substrate-PEO treated-polymer coated). The cytotoxicity of a range of drug concentrations (established from previous data), including the expected drug release concentrations (Figure 4b), was studied with murine endothelial cells (C166-GFP) and murine premyoblast cells (C2C12-GFP).

#### 3.3.1. Cytotoxicity of Effect of Released Paracetamol on Premyoblast and Endothelial Cells

**Figure 5:**
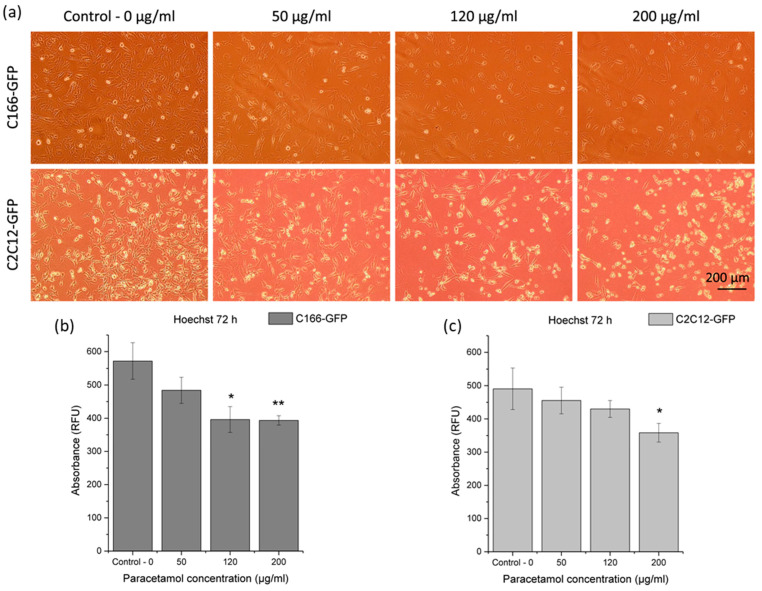
Paracetamol cytotoxicity: (**a**) Micrographs of C166-GFP and C2C12-GFP cells after 24 h in contact with different concentrations of paracetamol; (**b**,**c**) dsDNA quantification in cell cultures with different paracetamol concentrations after 24 h of treatment. Each condition is measured in relative fluorescence units (RFU) and compared with control without drug for each cell line. Significant differences stand for * (*p* ≤ 0.05), ** (*p* ≤ 0.01).

First, adherent cell cultures of premyoblast and endothelial cells were seeded on TCP substrates. Initially, all cell cultures showed a similar confluence level before paracetamol addition to culture media (48 h). Then, different drug dilutions were added to each triplicate, with increasing concentrations of paracetamol (0 µg/mL, 50 µg/mL, 120 µg/mL, and 200 µg/mL). Endothelial and premyoblastic cultures were photographed 24 h after drug addition (Figure 5a). As it can be expected, higher drug doses (specially 200 µg/mL) affected cell viability for both cell types: a lower number of adhered cells was found, and a higher number of detached cells and debris was observed. Nevertheless, all assayed drug dilutions showed living cells with proper cell morphology and adhesion to the plate.

DNA quantification (Figure 5b,c) confirmed previous observations concerning cell proliferation and viability (Figure 5a) for each concentration of paracetamol. DNA values, and therefore the number of cells, were significantly reduced at the highest drug concentrations for both cell models. In C166-GFP endothelial cells, 120 μg/mL condition was enough to induce a significantly lower DNA quantification signal than for the control condition (0 μg/mL); 200 μg/mL condition yielded a very significant difference. In contrast, using premyoblasts, the only condition with a significant reduction in cell concentration was observed for the treatment with 200 μg/mL of paracetamol.

The addition of paracetamol did not prevent the growth of endothelial cells and premyoblasts in the range of concentrations up to 120 μg/mL, so its presence in the biomaterial should not compromise the cell survival. If the proposed coating releases paracetamol in one burst, producing a local concentration of 120–200 μg/mL that is maintained for at least 24 h, it could affect cell viability according to the cytotoxicity results. However, PAR release from a complete Mg-HHC system showed a lower concentration range (Figure 4b), suggesting that paracetamol addition would not constitute a drawback in the biomaterial’s cytocompatibility. These observations, with respect to paracetamol cytotoxicity, do not have a clinical application by themselves since paracetamol is not administered locally. However, these results would enable us to discriminate cellular responses to the model implant that are due to the drug and the corrosion effect, and to find out whether the system offers enough control over the material degradation in the cell culture medium containing organic additives and cells. Fekry et al. have previously identified paracetamol as a corrosion inhibitor with respect to AZ91 Mg alloy in ethylene glycol and sodium chloride solutions [40]. However, Moreno has shown that paracetamol loaded into a PEO/PCL hybrid coating is a mild corrosion accelerator with respect to Mg-Zn-Ca alloy in modified α-MEM [28].

#### 3.3.2. Cytotoxicity Effect of Released Ciprofloxacin on Premyoblast and Endothelial Cells

**Figure 6:**
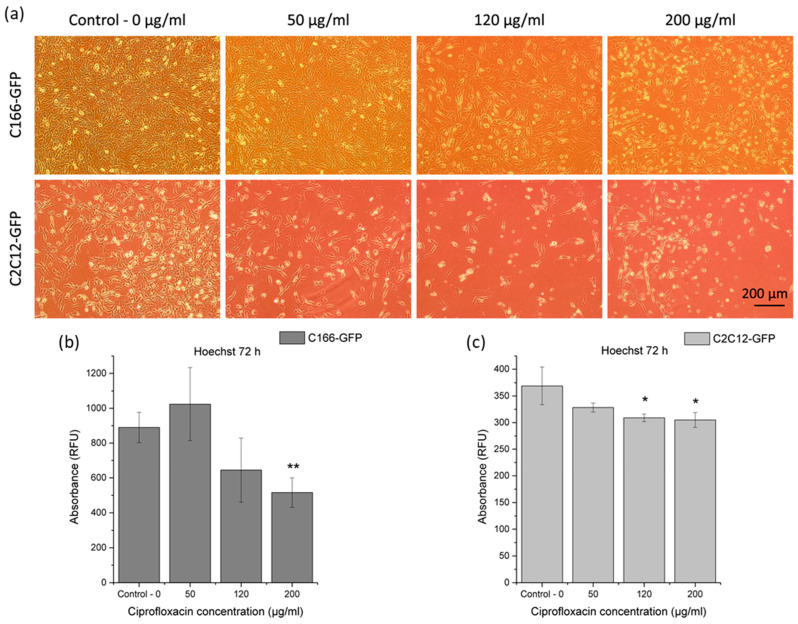
Ciprofloxacin cytotoxicity: (**a**) micrographs of C166-GFP and C2C12-GFP cells after 24 h in contact with different concentrations of ciprofloxacin; (**b**,**c**) dsDNA quantification in cell cultures with different ciprofloxacin concentrations after 24 h of treatment. Each condition was measured in relative fluorescence units (RFU) and compared with control without drug for each cell line. Significant differences stand for * (*p* ≤ 0.05), ** (*p* ≤ 0.01).

A similar protocol was used to test ciprofloxacin in vitro cytotoxicity in a TCP model. For both cell types, confluence was initially the same, but, after ciprofloxacin addition, higher doses clearly affected cell viability, showing lower confluent cultures with cell debris (Figure 6a). Likewise, drug addition did not totally disrupt the cell proliferation and plate adhesion in any condition.

Cell proliferation was also evaluated by total dsDNA quantification (Figure 6b,c). Regarding endothelial cells (C166-GFP), the condition with 200 μg/mL of ciprofloxacin showed a very significant reduction with respect to drug-free TCP control. In premyoblast cells assay (C2C12-GFP), total DNA values in 120 μg/mL and 200 μg/mL ciprofloxacin concentrations were significantly lower than those obtained in the control.

Ciprofloxacin assay produced similar results to those obtained with paracetamol in terms of cytotoxicity. Even in 50 μg/mL of ciprofloxacin condition, mean values were similar or higher than those in the control, but this difference was not statistically significant. For this reason, it is possible that ciprofloxacin at low concentrations (<50 μg/mL) enhances the cellular response, but this result could also have been obtained haphazardly. Studies of ciprofloxacin effect on cell cultures of other cell lines also observed a dose-dependent effect, and, at certain concentrations (~34 μg/mL), it could even significantly improve cell viability over the control; however, at higher doses, ciprofloxacin reduced viability by up to 60% [41].

### 3.4. Cytocompatibility of the Drug-Eluting External Layer with the Designed Topography

Once the effect of the drug concentration was evaluated, an analysis of cell interaction with the outer layer of the hybrid coating was performed. In this experiment, both surface topography and drug release may affect cell behavior. For this cytocompatibility study, BF-PCL drug-free and drug-loaded films over glass disks were used, including a control condition of cell growth on TCP.

Drug-free BF-PCL films were used to reveal how cells behave in relation to the coating topography. Only CIP-loaded BF-PCL films were evaluated as a representative model of drug-loaded coating since paracetamol and ciprofloxacin showed similar cytotoxicity. 

**Figure 7:**
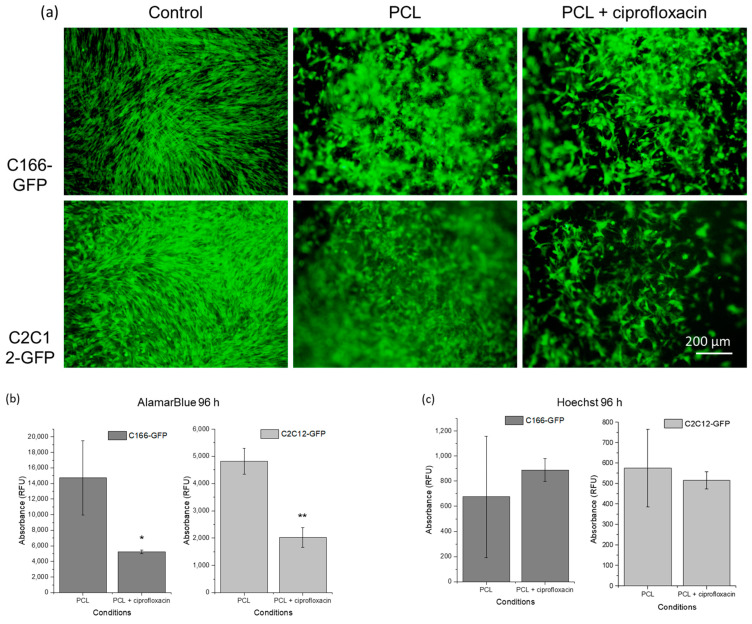
Cell growth on BF-PCL films and ciprofloxacin loaded BF-PCL films. (**a**) Fluorescence micrographs of C166-GFP and C2C12-GFP cell lines after 96 h of culture. Each condition is compared with control culture on TCP. Cytocompatibility assay with films: (**b**) Metabolic activity of the cells at 96 h; (**c**) Cell proliferation analysis by dsDNA quantification. For each cell line ciprofloxacin films results are compared with films without drug in both tests. Significant differences stand for * (*p* ≤ 0.05), ** (*p* ≤ 0.01).

After 96 h of cell culture, all samples showed viable cells proliferating over surfaces, but with differences. TCP control condition (Figure 7a) showed an almost confluent monolayer of cells, completely covering the TCP surface. In contrast, neither of the cell lines formed a dense monolayer on BF-PCL films, which may be related to porous topography and an increased surface culture area. In addition, slightly fewer cells are observed in films with ciprofloxacin than in the drug-free films. In all film samples, cells showed a prevalent adherent morphology, since almost no rounded or semi-adherent shapes were observed, but rather an extended cell morphology indicating interaction with the substrate. This effect occurred both in drug-free and drug-containing samples. As such, ciprofloxacin does not impede cell adhesion and proliferation on the surface, and topography achieved by breath figures sustains cell growth.

In order to quantitatively compare cell viability, a metabolic activity analysis was performed at 96 h of culture (Figure 7b). TCP controls of both investigated cell lines had metabolic activity values 3–6 times higher than those for control films (drug-free BF-PCL), being exclusively a positive control of the assay. These TCP controls were used only to confirm that cells were in good condition and the culture conditions were appropriate, so they were not included in the graphs (Figure 7b,c). Metabolic cell activity in both endothelial and premyoblast cell cultures (Figure 7b) is significantly lower (i.e., lower viable cell counts) in CIP-loaded films than in drug-free films. Further, viability of premyoblasts on films is lower than viability of endothelial cells, which may be due to the resistant and adaptable nature of endothelium.

Proliferation differences between cells on drug-loaded films and drug-free films were also estimated by dsDNA quantification at 96 h of cell culture (Figure 7c). None of the cases show significant differences between the films. The results indicate that while the drug affects the metabolic activity of the cells, partially reducing it, it does not significantly affect the cell proliferation.

### 3.5. Cytocompatibility of Mg-HHC System Loaded with Paracetamol or Ciprofloxacin

After testing cellular behavior in response to the outer layer of the biomaterial, three conditions of Mg-HHC material were tested (without drug, with paracetamol, and with ciprofloxacin) in endothelial cell culture. To study the effect that the implant could have on the surrounding tissue, cells were seeded on the surface of the material and photographs were taken at different times, and cell proliferation and viability were quantified at 96 h of culture.

**Figure 8:**
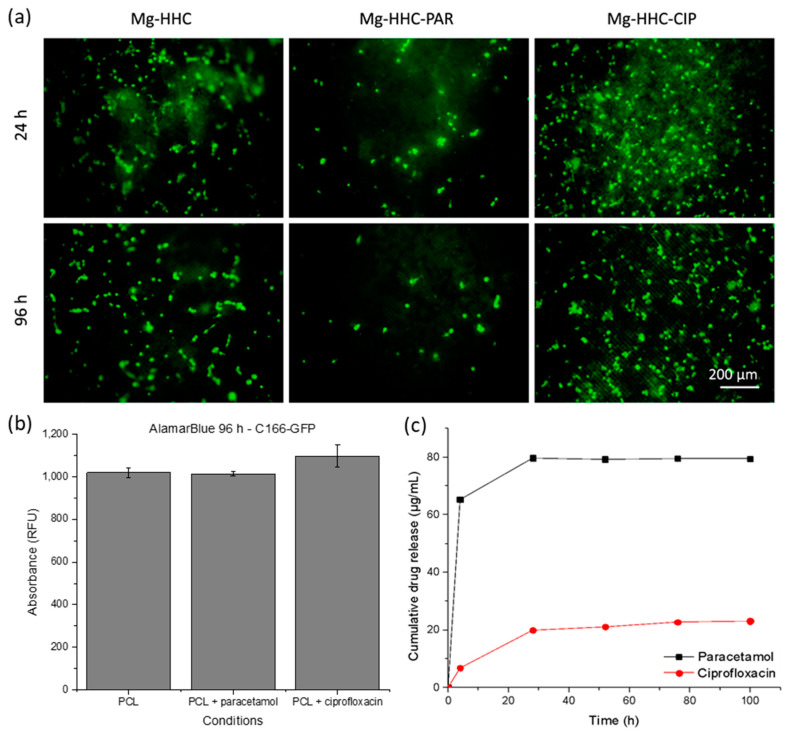
Cytocompatibility of the complete hybrid hierarchical coating loaded with paracetamol and ciprofloxacin. (**a**) Cell growth on Mg/PEO/Sealing-PCL/BF-PCL loaded with drugs, fluorescence micrographs of C166-GFP after 24 h and 96 h. (**b**) Cell viability on the complete system. (**c**) Drug release from Mg-HHC samples during 96 h of C166-GFP cell culture. Immersed in DMEM, incubated at 37 °C for 100 h: 4 h pre-treatment and 96 h cell culture.

Figure 8a shows that cell cultures remain similar in appearance from 24 h to 96 h. 

Cell density on the coatings did not seem to vary with time. Cell morphology also does not differ with time, conserving a rounded morphology, indicative of a poor adhesion to the substrate. At 96 h, fluorescence images of Mg-HHC-PAR samples show fewer cells than images of drug-free samples. In contrast, in Mg-HHC-CIP samples this situation is not so clear, and there are fields with similar or even higher proliferation in comparison with the controls. Therefore, cell culture responded poorly, i.e., they have not been able to reach an extended morphology and continue their cell cycle, inhibiting proliferation on the material. These deteriorated cell cultures showed low absolute values of metabolic activity with no significant differences between them (Figure 8b). Fewer cells are observed in samples with paracetamol than in drug-free samples. 

Considering that a stand-alone BF-PCL (i.e., the material of the top layer of Mg-HHC) allowed for good cell growth and that the loaded drugs did not prevent cell adhesion and proliferation, the effect of decreased cytocompatibility is probably caused by an increased local pH due to the corrosion of PEO-coated Mg alloy. Further, the addition of paracetamol had a visible effect on cellular response because paracetamol is known to enhance the corrosion process of PEO-coated Mg-Zn-Ca alloy generating an environment with even higher pH. 

Drug release from the complete system samples evaluated throughout cell culture assays (Figure 8c) explains the negative paracetamol effect; after 30 h immersion, there is considerably more free PAR in the medium (80 µg/mL) when compared with the Figure 4b data (40 µg/mL), i.e., the release kinetics is faster. The contrary is observed for CIP-loaded Mg-HHC. This may be related to the effect of amino acids and FBS protein in the cell culture medium, as well as the presence of the cells. The CIP uptake and retention by the cells may be the reason for the lower amount of free CIP detected in the medium.

### 3.6. Coating Performance of the Mg-HHC System in Cell Culture

To evaluate the performance of the coating and the influence of Mg degradation process on cellular response to the material, the specimen morphologies were analyzed pre- and post-cell culture. The initial Mg-HHC degradation that occurs during 4 h of sterilization process and sample preparation as well as the subsequent degradation that takes place during 96 h of cell culture were evaluated. 

#### 3.6.1. Cell Response to the Drug-Eluting Hierarchical Hybrid Coating

**Figure 9:**
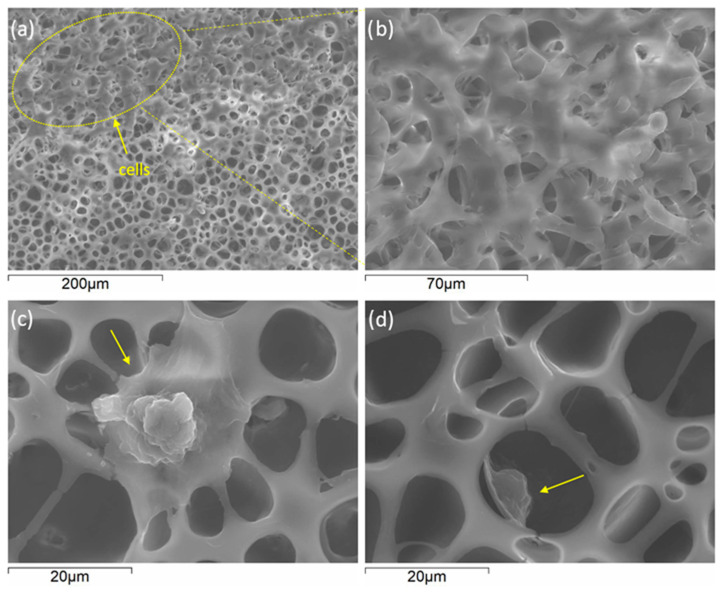
SEM micrographs of C166-GFP cells after growing 96 h on Mg-HHC-CIP. (**a**–**c**) Cells growing over the BF-PCL top layer loaded with ciprofloxacin, (**d**) cell inside the pores of drug-free outer BF-PCL layer. Cells are indicated by yellow arrows.

Cellular response was studied in plan view SEM micrographs of the three types of sample preparations. In Figure 9, cells with partially extended morphology are observed on the coating, noticeable in image (c), in addition to some cell penetration inside the coating pores. In general, a low number of cells is observed on the surface of all samples, which is in good agreement with previously reported data. Figure 9a,b reveal a localized area of CIP-loaded coating with a higher cell density, in which cells are partially extended on the surface and others colonizing more internal areas of the coating. Ciprofloxacin release may have helped to create a less aggressive microenvironment [32] because it tends to form insoluble complexes with Mg and Ca that precipitate and block the defects in the coating. Therefore, it promoted the survival and proliferation of cells in the area.

In the CIP-free coating (Figure 9d), cells appear to colonize inner regions of the pores in the BF-PCL layer. This suggests that a 3D structure of the coating topography could be beneficial for cell adhesion and proliferation.

#### 3.6.2. Degradation of the Mg-HHC during Sample Preparation for Cell Culture

**Figure 10:**
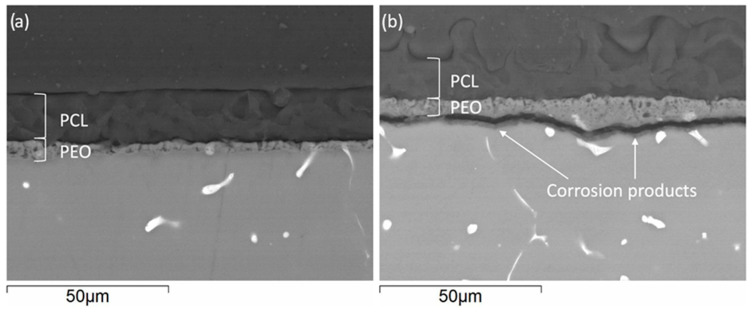
Backscattered electron cross-sectional micrographs of (**a**) Mg-HHC and (**b**) Mg-HHC + CIP after 4 h of incubation in DMEM at 37 °C.

The cross-section images of drug-free and CIP-loaded Mg-HHC systems after 4 h of immersion in DMEM are illustrated in Figure 10. The corrosivity of DMEM is defined by inorganic components in its composition (6.4 g/L NaCl, 3.7 g/L NaHCO_3_, 0.4 g/L KCl, 0.109 g/L Na_2_HPO_4_, 0.09767 g/L MgSO_4_, 0.2 g/L CaCl_2_, 0.0001 Fe(NO_3_)_3_·9 H_2_O). The non-loaded Mg-HHC system remains without signs of degradation after 4 h of incubation in the cell culture medium. However, the presence of a thin layer of corrosion products under PEO coating was observed in some locations in the case of Mg-HHC + CIP. It should be noted that the macro-appearance of the specimens was intact, with no detectable signs of degradation, so the cross-sectioning was performed entirely arbitrarily. 

This suggests that the corrosion process may be starting during the incubation process in both loaded and drug-free specimens and the H_2_ release would already be occurring at the time of cell seeding. The pH alkalization would make it difficult for cells to adhere properly to the surface. 

#### 3.6.3. Characterization of the Mg-HHC System after 96 h of Cell Culture

**Figure 11:**
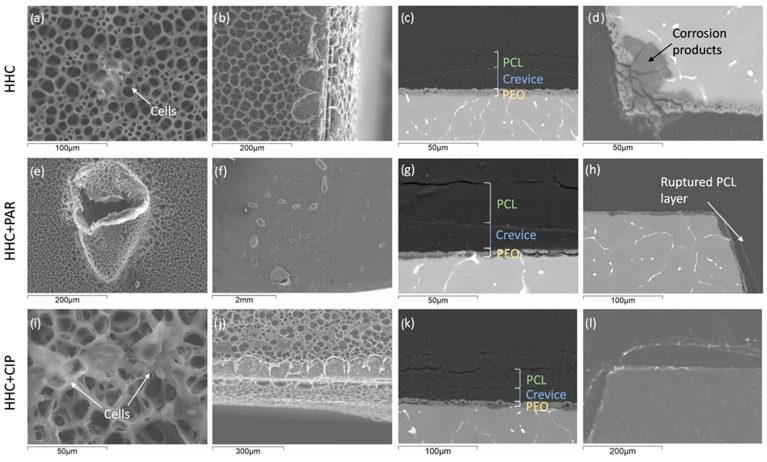
(**a**,**b**,**e**,**f**,**i**,**j**) Secondary electron plan views and (**c**,**d**,**g**,**h**,**k**,**l**) backscattered electron cross-section micrographs of (**a**–**d**) non-loaded, (**e**–**h**) PAR-loaded and (**i**–**l**) CIP-loaded Mg-HHC systems after 96 h of immersion in cell culture conditions.

In order to verify the development of corrosion mechanism of the specimens, non-loaded and loaded Mg-HHC systems have been analyzed in more detail after 96 h of immersion in cell culture medium (Figure 11). In depth analysis of SEM micrographs allows us to understand the phenomena responsible for the obtained cellular response.

There are cells both at the surface of the samples and inside the pores of the BF, as is the case of Mg-HHC and Mg-HHC + CIP systems (Figure 11a,i). As discussed earlier, the higher concentration of cells at some locations may be associated with the lowest aggressive microenvironment in these areas, facilitating the cell adhesion and proliferation. The bulk pH was measured during the experiment in each well, and the pH for all specimens (8.25–8.4) was similar to that of the pre-culture medium (pH 8.2). Note that all the pH measurements were conducted at room temperature, since the medium in 6 mL well cools down rapidly once out of the incubator. It is likely that the notable increase in pH occurs in the microenvironment of the surface vicinity, thus affecting the cell viability. However, in the total volume of the well, the effect of alkalization becomes diluted as the pH change can be compensated by the CO_2_ buffer of the incubator system.

Importantly, the presence of defects in BF PCL layer at the edges of the samples are observed (Figure 11b,j). These can be attributed due to the concentration of stresses around the edges caused by the evaporation of the chloroform and the solidification of the PCL. These defects induce corrosion process and localized formation of thick corrosion products, facilitating the propagation of the crevice at the PCL/PEO interface. Edges and corners are the main areas where coatings fail (Figure 11d,h).

Corrosion products at the Mg/PEO interface are detected in all conditions, with an active corrosion process under PEO layer. For all samples, the presence of crevice at PEO-PCL interface (i.e., PCL coating lift-off) is observed (Figure 11c,g,h). It is likely that the crevice initiates at the PCL sealing/BF-PCL interface because, in the high humidity of the chamber where the BF top-coat is formed, the hydrophobic behavior of PCL sealing layer allows the water droplets to accumulate on the surface of the sample, compromising the adhesion between the two polymer layers. The development of thick corrosion products layer occurs in the area of the crevices. From the exposed corner, the DMEM media can penetrate and propagate through these crevices. As such, the combined effect of corner/edge defects in BF-PCL top-coat and adhesive failure between the two polymeric layers enhance corrosion throughout the material.

All specimens show the formation of blisters on the surface of the sample, being more pronounced in the case of Mg-HHC + PAR (Figure 11e,f). The formation of blisters is associated with the accumulation of H_2_ gas in the PEO/Mg interface caused by the degradation of Mg. The corrosion inside the PCL blisters is more intense (note the corrosion products at PEO/Mg interface, Figure 11c,g,k because the microenvironment is more aggressive due to the increase of Cl^−^ concentration. The local pH inside the intact blister may become lower than 7.4 as a result of Mg(OH)_2_ production. When the polymer layer does not rupture, these bubbles would act like a crevice, accelerating the corrosion in that area. The accumulation of H_2_ gas at the interface leads to the stretching of PCL due to its high flexibility [42], and the blister grows until a rupture occurs. Note a perforated blister of about 300 μm in length in the BF-PCL layer (Figure 11e). This defect facilitates the contact of the culture medium with the inner layers. Blister formation is consistent with a particularly low cell density in PAR-loaded system, possibly as a result of the corrosion-accelerating effect of this drug. The observed decrease in cytocompatibility is probably due to an increase in local pH of the material (up to 0.2 pH units increase in the bulk with respect to the pre-culture medium) caused by corrosion of the magnesium alloy.

**Figure 12:**
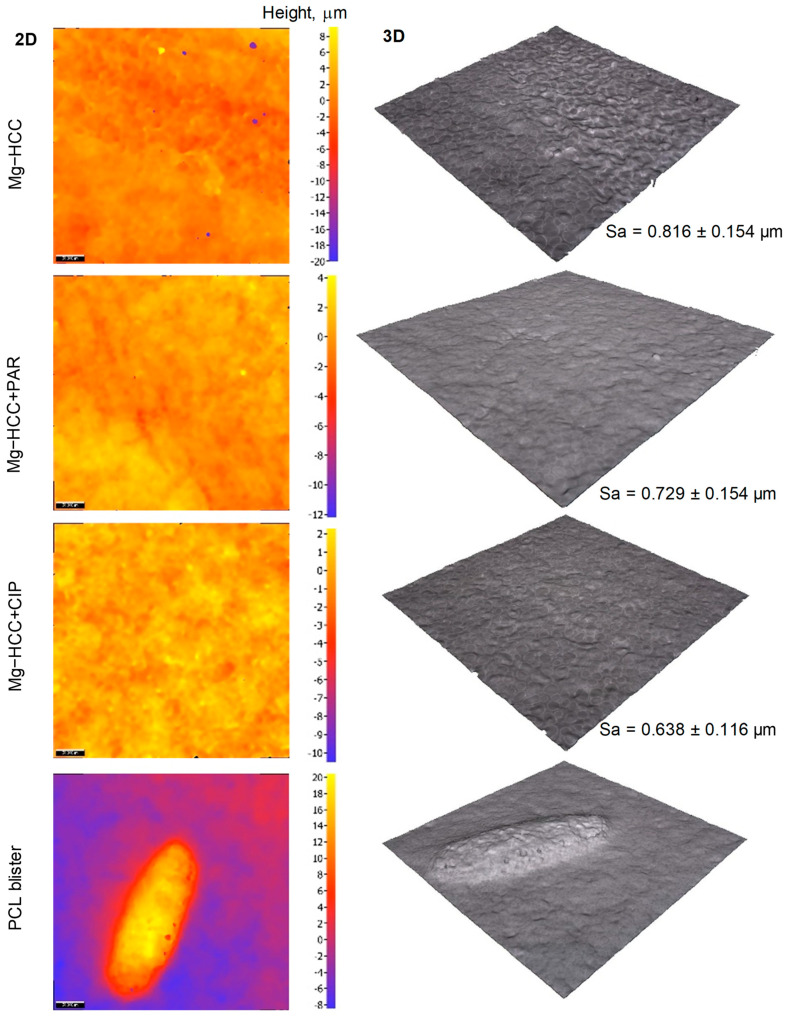

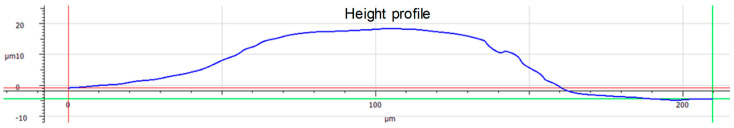
Optical profilometry micrographs of non-loaded and loaded HHC system coating after 96 h of cell culture. Topography 2D images (**left**), 3D-rendering of the surface (**center**) and arithmetical mean height of the area “S_a_” (**right**). Height profile of a blister (**bottom**).

**Figure 13:**
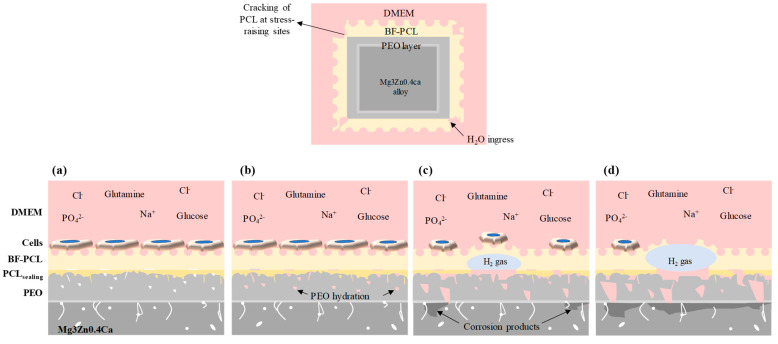
Schematic of the Mg-HHC system degradation: (**a**) 1–4 h of incubation; (**b**–**d**) 4–96 h of incubation.

The S_a_ values of the specimens (Figure 12) measured in blister-free areas showed no significant differences between samples after the cell culture medium, which may be because the BF pores constitute the main feature of the surface topography. However, differences were observed in S_a_ before and after the test (2.54 ± 0.47 μm and 0.82 ± 0.15 μm, respectively). In areas where blisters were present, the average blister was ≥20 μm in height and ≥100 μm in length. In some cases, corrosion products are locally formed at the confluence sites of the secondary phases, which are present at the Mg/PEO interface (Figure 11c,d,g). Once the medium passes through the crevice at the interface of PCL layer and hydrates the porous PEO layer (Figure 13), it comes in contact with the secondary phases of the alloy, through micro-crevices formed in the PEO coating, which is in agreement with observations of Moreno et al. [43]. Secondary phases are rich in Zn, which implies strong galvanic micro-couples with the α-Mg matrix. The corrosion process at these sites is enhanced and generates a lot of H_2_, leading to local pH increase and negatively affecting cell survival.

## 4. Conclusions

It may be concluded that the biodegradation of the developed Mg-HHC system comprising a ceramic PEO layer, a sealing PCL layer and a breath figure PCL top-layer initiates according to the following mechanism, outlined in Figure 13: (i) the penetration of the corrosive species from the media through the edge and corner defects in polymer layer, (ii) formation of a crevice at PCL_sealing_/BF-PCL interface, (iii) the hydration of PEO coating by the penetration of the media through pores and cracks in PEO; (iv) PEO barrier layer failure with subsequent formation of undercoating corrosion products; (v) formation of microgalvanic couples between nobler intermetallic inclusions at the PEO/Mg interface and the Mg matrix, accelerating the corrosion process. As a consequence of the latter, the formation of stretched PCL blisters causes a more aggressive Cl^−^ concentrated microenvironment inside, further promoting the localized corrosion mechanism. The accumulation of more H_2_ gas allows the PCL bubble to grow causing mechanical stress, delamination of the polymer layer and eventual bursting of the blisters which facilitates further ingress of corrosive medium towards the coating/substrate interface. 

BF-PCL top-coat of the HHC systems provides a porous topography that enhances cytocompatibility and ensures drug release, while affecting the local microenvironment. The developed Mg-HHC system presents interest for further adaptation, e.g., via modification of the layer assembly and refinement of the fabrication, to different pharmaceutical agents and development of new therapy strategies, including cancer therapy. 

## Figures and Tables

**Table 1:** Dip-coating conditions for the fabrication of polymeric layers of HHC.

Coating Layer	Withdrawal Speed	No. of Immersion Cycles
Sealing PCL	0.3 mm/s	1
BF PCL	2 mm/s	2

## Data Availability

Data available in a publicly accessible repository that does not issue DOIs. All data presented in this work will be available in Docta Complutense repository https://docta.ucm.es/handle/20.500.14352/16 from 17 December 2023.
